# Use of Silicone Sizers in Implantation of Porous Polyethylene Nasal Dorsal Implants in Asians

**DOI:** 10.1155/2011/606941

**Published:** 2011-09-20

**Authors:** Randal Pham

**Affiliations:** Aesthetic & Refractive Surgery Medical Center, 455 O'Connor Drive, Suite 180, San Jose, CA 95128, USA

## Abstract

*Introduction*. A new technique of implantation of high-density porous polyethylene nasal dorsal implants in Asians is described in this paper. Silicone sizers, which have smooth surfaces, were used to facilitate implantation of porous polyethylene implants in Asian patients. *Materials and Methods*. Twenty-three patients of Asian descent underwent dorsal augmentation rhinoplasty with open technique using high-density porous polyethylene implants. In all cases, silicone sizers were used to facilitate implantations of high-density porous polyethylene nasal dorsal implants. Patient selection criteria exclude patients with history of cocaine use, history of nasal or sinus disorders, previous nasal surgery, deviated septum, poor cartilage support, and thin skin. *Results*. No bleeding, infection, rejection, displacement, or extrusion was noted. One implant was removed because of a patient's dissatisfaction with the resulting tip height. * Conclusion*. The use of silicone sizers to facilitate implantations of high-density porous polyethylene nasal dorsal implants was safe and efficacious.

## 1. Introduction

A new technique of implantation of high-density porous polyethylene nasal dorsal implants in Asians is described in this paper. Successful use of porous polyethylene implants to augment nasal dorsum of Asians and other races was previously reported [1–5]. The rough surface of porous polyethylene implants presents challenges to implanting this alloplastic material in Asian patients, who typically have small and short columella. In this paper, use of silicone sizers, which have smooth surfaces, to facilitate implantation of porous polyethylene implants in Asians is described. A review of medical literature showed that this paper is the first to describe such technique.

## 2. Materials and Methods

Charts of twenty-three patients of Asian (Chinese, Filipino, Cambodian, and Vietnamese) ancestries, one male and twenty-two female patients with age ranging from 20 to 57 years old, who underwent dorsal augmentation rhinoplasty with open technique using high-density porous polyethylene implants (Petite Nasal Dorsal Implants, manufactured by Stryker CMF, Newnan, Georgia, USA) from 2003 to 2011 were reviewed. The average length of follow up was 36 months. In all cases, silicone sizers (manufactured by Stryker CMF, Newnan, USA) were used to facilitate implantation of porous polyethylene nasal dorsal implants. Patient selection criteria exclude patients with history of cocaine use, history of nasal or sinus disorders, previous nasal surgery, deviated septum, poor cartilage support, and thin skin.

## 3. Selection of Implant Size

The size of the implant to be used in patients was selected based on the measured length of distance between the nasion and the tip-defining point and the width of the dome. The dorsum lengths, the lengths of the distance between the nasion and the tip-defining point of patients' noses, were measured. The lengths of the implant were selected to match the dorsum lengths. The dorsum widths, the widths of the dome of the noses (the distance between the domes of the medial crura), were used to select the dorsum widths of the implant. The implant widths were selected to be larger than one-third but smaller than two-third of the patients' dorsum widths. Implants were trimmed with a number 15 Bard Parker blade scalpel at the cephalic ends or on the sides to meet the selection criteria before implantations. The use of the silicone sizers intraoperatively further refined the selection process.

## 4. Surgical Technique

**Figure 1:**
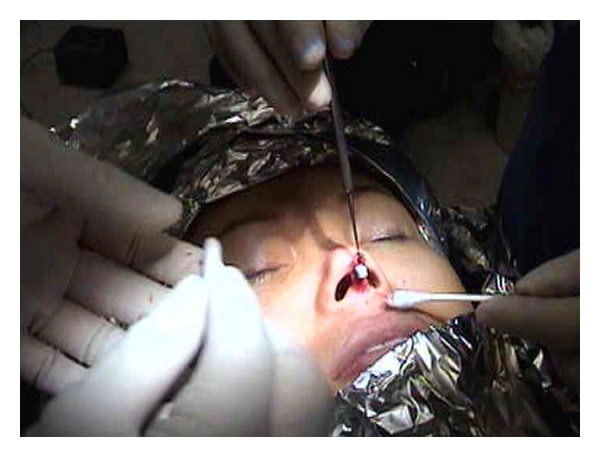
The silicone sizer (blue color) was pulled out one third of its length to provide adequate space for insertion of porous polyethylene implant.

**Figure 2:**
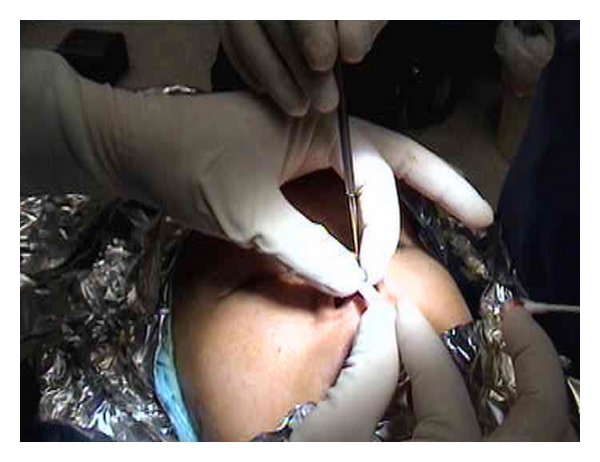
The porous polyethylene implant was inserted below the silicone sizer.

Surgery was performed in a private suite under local anesthesia. Approximately 5 mL of 1% lidocaine with 1 : 200,000 epinephrine was infiltrated into the columella and along the dorsum of the nose from the tip of the nose to the nasion. A horizontal incision was made with a number 15 Bard Parker blade scalpel along the base of the columella at the columellar-labial junction. Marginal incisions on both sides of the columella were made 2 mm behind the leading edge of the columella. The incision was then carried superiorly along the mucocutaneous junction to the soft triangle. Dissection of the columellar skin over the medial crura, and laterally was done with tenotomy scissors. Care was taken not to damage the medial crura and dissection was stopped short of the dome. The angle of dissection was changed from parallel to the medial crura to parallel to the dorsum of the nose. Blunt dissection below the musculoaponeurotic layer was performed from the caudal end of the medial crura through the tip, and the supra tip, the dorsum and stopped at the nasion. A single hook was used to expose the lower lateral cartilage. During blunt dissection, an effort was made to stay below the periosteum of the nasal bones and at the same time to avoid following the upper lateral cartilage below the caudal margin of these bones. A sizer of the selected implant was inserted. The skin was draped over the sizer and was evaluated for adequate thickness to cover the implant with no excessive tension placed over the tip area. The sizer was then pulled out one third of its length to provide adequate space for insertion of the porous polyethylene implant (Figure 1). The selected porous polyethylene implant was inserted below the sizer and slid in as the sizer was removed (Figure 2). The porous polyethylene implant was checked for tip tension and accurate positioning and adjusted with a sterile Q tip accordingly. The skin wound was closed with 6–0 plain gut sutures in interrupted manner at the columellar-labial junction and along the lateral margins of the columella. A cast was placed over the nasal dorsum to secure the implant.

## 5. Results

The average length of followup was 36 months. No bleeding, infection, rejection, displacement, or extrusion was noted. One implant was removed because of a patient's dissatisfaction with the resulting tip height. The patient would like to have a higher projection. The author refused to reimplant a larger implant. The patient found a cosmetic surgeon who agreed to implant a larger implant. The original implant was removed by the author without complication.

## 6. Discussion

Since this author reported the safety and efficacy of the use of high-density porous polyethylene implants for dorsal augmentation rhinoplasty in Asians [1], several authors have reported successful uses of the implants in the nose [6–15]. The rough surface of porous polyethylene implants, however, presents a challenge to surgeons who wish to minimize trauma to the subcutaneous tissue of the tip and dorsum of the nose during implantation of this alloplastic material. The friction created by the rough surface of porous polyethylene implants can cause damage to the musculoaponeurotic layer during insertion of the implants. The use of the silicone sizers provides a guiding passage for surgeons to insert with ease the porous implants, which have a relatively rougher surface compared to that of the silicone sizers.

The implantation becomes more difficult when the technique is used on Asians, who typically have small nose with short columella. The use of silicone sizers allows surgeons to evaluate tip tension before implanting porous polyethylene implants. One patient desired a larger implant after surgery; the author recognized the potential complications a larger size implant could cause, in this case, extrusion with increased possibility of infection, and declined to re-implant a larger implant.

In summary, the pores of the porous polyethylene implants, which allow vascularization and eventual migration of fibrovascular tissues into these implants [1–5], cannot be modified into the completely smooth surface characteristic of silicone implants. However, with increased vascularization and tissue migration into the pores, infection and extrusion can be prevented; this characteristic of porous polyethylene implants is a clear advantage over silicone implants [15]. The use of the silicone sizer facilitates implantation of porous polyethylene implants and provides patients with all the benefits that porous polyethylene implants offer.

## 7. Conclusion

It was safe and efficacious to use silicone sizers to facilitate implantations of high-density porous polyethylene nasal dorsal implants in Asian patients.
